# Effect of Arabinoxylan from Wastewater Generated during Vital Wheat Gluten Production on Liver Metabolism in Type 2 Diabetic Mice

**DOI:** 10.3390/foods12142640

**Published:** 2023-07-08

**Authors:** Denglin Luo, Xingguo Li, Mengyuan Geng, Yunhui Zhang, Honglin Lan, Jiale Li, Caili Qi, Zhouya Bai, Jihong Huang

**Affiliations:** 1 Food and Pharmacy College, Xuchang University, Xuchang 461000, China; 2 Henan Engineering Research Center of Food Material, College of Food and Bioengineering, Henan University of Science and Technology, Luoyang 471023, China

**Keywords:** AX, type 2 diabetes, liver metabolism, UHPLC-MS/MS

## Abstract

Arabinoxylan (AX) is a dietary fiber that has been proven to have a significant antidiabetic effect. Liver metabolic disorders frequently coincide with the development of type 2 diabetes, but research on the hepatoprotective effects of AX in type 2 diabetic mice is lacking. As AX is abundant in the wastewater produced during vital wheat gluten protein production, this study used it as a raw material to evaluate its protective effect on liver function. The study employed an AX intervention in type 2 diabetic mice induced by a high-fat diet combined with streptozotocin and collected serum and liver tissue samples after 4 weeks. Serum and liver function indicators were measured using an automatic biochemistry analysis apparatus, and liver fat accumulation was observed using oil red O staining. Nontargeted metabolomics analysis of liver tissues was conducted using UHPLC-MS/MS. The results showed that AX significantly improved liver function indicators and histopathological damage, and regulated liver metabolic disorders by improving the differential metabolites of pantothenate and CoA biosynthesis, as well as purine metabolism. This study demonstrated that AX may exert a significant hepatoprotective effect by regulating metabolic disorders.

## 1. Introduction

With the ongoing enhancement of people’s living standards, diabetes has become more prevalent [1]. The escalating prevalence of diabetes and the concomitant surge in healthcare expenditures impose a colossal burden on global systems related to society, finance, and health [2]. According to research, it was estimated that 10.5% of individuals aged 20–79 worldwide had diabetes in 2021, which means a total of 536.6 million people were affected. This percentage is expected to increase to 12.2%, or approximately 783.2 million people, by the year 2045. In terms of healthcare costs, in 2021, the worldwide expenditure for health issues associated with diabetes was estimated at 966 billion USD; moreover, this figure is estimated to escalate to 1045 billion USD by 2045 [3]. Type 2 diabetes is a prevalent metabolic disorder with a multifactorial pathogenesis, accounting for over 90% of all diabetes cases [4,5]. Currently, the drugs commonly utilized in clinical practice for the management of type 2 diabetes comprise biguanides, sulfonylureas, and enzyme inhibitors. These drugs can achieve a certain hypoglycemic effect, but long-term use can have side effects on the body, such as poisoning, gastrointestinal discomfort, and hypoglycemia [6]. Therefore, there is a need for innovative therapeutic methods utilizing natural resources and dietary intervention due to their high effectiveness, minimal side effects, and low toxicity.

Dietary fiber is a type of carbohydrate present in plant-based foods, primarily composed of polysaccharides that cannot be digested and absorbed by the human body [7]. It encompasses various forms, such as cellulose, hemicellulose, and soluble fiber. Dietary fiber plays a crucial role in the human digestive process, including improving digestive system function, stimulating intestinal hormones such as glucagon-like-peptide-1 (GLP-1), regulating blood sugar levels, reducing cholesterol levels, and promoting cardiovascular health [8,9,10], among others. These beneficial effects make dietary fiber one of the most important factors in preventing and improving diabetes. Exploring the antidiabetic activity of dietary fiber through dietary supplementation has become a current research hotspot.

Arabinoxylan (AX), as a natural dietary fiber and as a dietary fiber, AX has been proven to possess numerous physiological and pharmacological benefits [11], including enhancements in glucose metabolism [12], antitumor activity [13], modulating gut microbiota [14,15], and immune regulatory activity [16]. At the same time, the rise and popularity of metabolomics have provided new ideas for the study of type 2 diabetes, greatly promoting in-depth research on the antidiabetic effects of AX. Studies have demonstrated that AX can ameliorate disorders in the TCA cycle, tryptophan metabolism, lipid and ketone body metabolism, taurine and hypotaurine metabolism, as well as lysine metabolism, in rats with type 2 diabetes [17]. It regulates diabetes-induced dyslipidemia by adjusting the levels of metabolites related to dyslipidemia [18]. It also alleviates type 2 diabetes by enhancing the composition of bacterial populations such as *Actinobacteria*, *Proteobacteria*, *Escherichia-Shigella*, and *Klebsiella* in the gut [19]. The liver plays a critical role in the metabolic pathways of both carbohydrates and lipids [20,21]. Abnormal liver function is commonly associated with the development and worsening of diabetes, and a compromised liver state may impair the function and survival of pancreatic beta cells, as well as peripheral insulin resistance [22]. AX can activate lipid metabolism by enhancing the activity of enzymes such as lipases in the liver and reduce oxidative stress in the liver by improving the level of superoxide dismutase, thereby alleviating liver damage [23]. However, there is limited research on the regulatory effects of AX on type 2 diabetes liver metabolism, and its protective effects on the liver remain to be explored.

Wheat contains a significant amount of nonstarch polysaccharides, with the majority being AX, aside from starch and gluten [24]. According to statistics, the production of vital gluten protein generates 24 million tons of starch wastewater annually. Although this wastewater is not toxic, the high viscosity makes it challenging to process using conventional sewage treatment techniques, and direct discharge would inevitably lead to environmental pollution [25,26]. As such, processing the wastewater from vital gluten protein production can serve as an excellent source of AX. Additionally, the effective utilization of this wastewater can play a crucial role in improving factory economic benefits and mitigating the issue of sewage discharge [25].

The objective of this research was to develop an effective approach for the separation and purification of AX from wastewater produced during the production of vital gluten proteins. Additionally, untargeted metabolomic techniques based on UHPLC-MS/MS would be employed to investigate the effects of AX on hepatic metabolism in mice with type 2 diabetes induced by a high-fat diet and streptozotocin. The goal was to uncover the potential value of AX and provide inspiration for the development of treatment methods that were appropriate for patients with diabetes.

## 2. Materials and Methods

### 2.1. Preparation of AX

The wastewater (produced by Henan Feitian Agriculture Development Co., Ltd., Hebi, China) was centrifuged at 4000 rpm for 15 min, and the sediment was discarded after centrifugation. Then, the supernatant was obtained and condensed into one-fourth of the original volume by a rotary evaporator. Anhydrous ethanol was added to achieve an ethanol concentration of 80%, followed by overnight settling and centrifugation at 4000 rpm for 15 min. The sediment was dissolved in water, and the resulting solution was again concentrated by rotary evaporation. After dialysis for 24 h, the solution was vacuum freeze-dried to obtain crude AX. The pretreated DEAE-cellulose was placed in a shaker bottle, and a solution of crude AX with a concentration of 10 mg/mL was added (DEAE-cellulose and AX crude solution were mixed in a ratio of 1:5, *w*/*v*). The shaker bottle was then sealed, and the solution was adsorbed for 6 h under conditions of 120 r/min at 25 °C. After filtration, a decolorized and deproteinized polysaccharide solution was obtained. The solution was centrifuged (5000 r/min, 10 min), and four times the volume of the supernatant was added with 95% ethanol to induce precipitation. The sediment was solubilized in water, followed by the removal of ethanol via rotary evaporation. The resulting sample was vacuum freeze-dried to obtain purified AX with a purity greater than 80%. The detailed information on AX is available in Supplementary Table S1.

### 2.2. Animal Experiment

C57BL/6J male mice, 6 weeks old and weighing an average of 14 g, were purchased from Beijing Sibeifu Bioscience, Co. Ltd. (Beijing, China). All mice were adapted to one week of rearing under controlled conditions with a temperature range of 21 ± 2 °C and a light-dark cycle of 12/12 h, during which time they were fed standard feed. After one week, five mice were used as a control (CON) group to maintain a normal standard diet, while the remaining mice were fed a high-fat diet. Following a 12-h fasting period at the 4-week mark, mice that had been fed a high-fat diet were intraperitoneally administered streptozotocin at a dosage of 120 mg/kg body weight (BW), which resulted in the onset of type 2 diabetes. One week later, the mice were evaluated for diabetes based on their fasting blood glucose (FBG) levels, which were considered to be indicative of diabetes if they exceeded 11.1 mmol/L.

**Figure 1:**
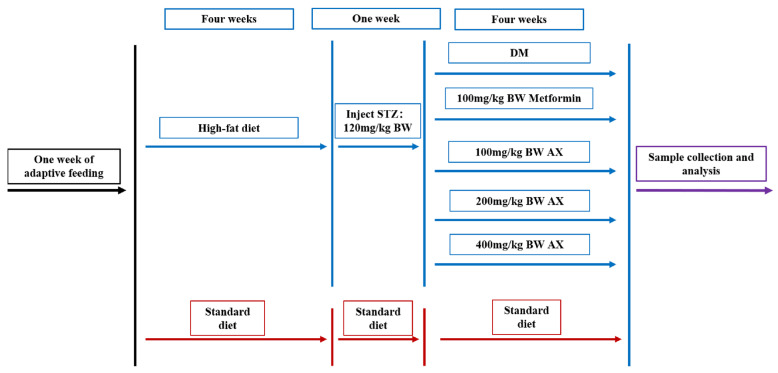
Design of animal experiments. STZ: streptozotocin, DM: diabetes mellitus, AX: arabinoxylan, and BW: body weight.

After that, the control group maintained a standard diet and a high-fat diet with diabetic mice, respectively. Five groups of diabetic mice were formed through random division (*n* = 5); diabetes mellitus (DM) group; metformin (Met) group: 100 mg/kg BW metformin gavage daily; low-, medium-, and high-dose AX groups (AX100, AX200, and AX400): 100, 200, and 400 mg/kg BW AX gavage daily, respectively. The control group, DM group, and Met group consumed the same amount of pure water every day. The experimental design was presented in Figure 1.

### 2.3. Sample Collection

Following a 4-week treatment period, the mice were euthanized through eye extraction while under anesthesia induced by isoflurane after fasting. Blood samples were collected and allowed to stand at room temperature for half an hour. Subsequently, the samples were subjected to centrifugation at a speed of 3000 rpm for 15 min in order to obtain serum for biochemical analysis. The liver tissues were extracted and preserved at −80 °C for subsequent analysis. All animal experiments were performed in strict accordance with the international rules and ethical principles for the use and care of laboratory animals, and were approved by Spife (Beijing, China) Biotechnology Co., Ltd. [SCXK (Jing) 2019-0010].

### 2.4. Serum Liver Functional Indicators Analysis

An automatic biochemistry analysis apparatus was utilized to determine the levels of aspartate aminotransferase (AST), alanine aminotransferase (ALT), total protein (TP), and albumin (ALB) in serum.

### 2.5. Histological Analysis

Liver tissue was frozen sectioned at 8 μm and fixed with 10% neutral formalin for 10–15 min, followed by washing with water. The sections were then stained with freshly prepared Oil Red O for 10–15 min, and were subsequently decolorized with 60% isopropanol. After being rinsed with distilled water, the nuclei were lightly stained with hematoxylin, and the sections were mounted with a glycerin-gelatin mounting medium. The liver lipid changes were observed, and images were collected under a microscope at 400× magnification.

### 2.6. Metabolomics Analysis of Liver Tissue

To prepare the sample for analysis, a weight of 25 mg was placed into an Eppendorf tube and combined with 500 μL of extract solution comprising methanol and water in a 3:1 ratio, along with a mixture of internal standards labeled with isotopes. Subsequently, the samples were homogenized at a frequency of 35 Hz for a duration of 4 min, followed by sonication in an ice-water bath for 5 min. This process was repeated thrice. After incubating for an hour at −40 °C, the samples were subjected to centrifugation at 12,000 rpm for 15 min at 4 °C to obtain a supernatant, which was transferred to a fresh vial for further analysis. Additionally, the quality control (QC) sample was created by combining equivalent volumes of the supernatants from each sample.

For the LC-MS/MS analyses, an UHPLC system (Vanquish, Thermo Fisher Scientific, Waltham, MA, USA) was utilized, which was coupled with an Orbitrap Exploris 120 mass spectrometer (Orbitrap MS, Thermo) and a UPLC HSS T3 column (2.1 mm × 100 mm, 1.8 μm). The mobile phase comprised a mixture of 5 mmol/L ammonium acetate and 5 mmol/L acetic acid in water (A) and acetonitrile (B). The auto-sampler temperature was set at 4 °C, and the injection volume was 2 μL. The Orbitrap Exploris 120 mass spectrometer was selected for its ability to obtain MS/MS spectra in information-dependent acquisition (IDA) mode, under the control of the acquisition software (Xcalibur, V4.4, Thermo), which continuously evaluated the full-scan MS spectrum.

### 2.7. Statistical Analysis

GraphPad Prism 9.5.0 (GraphPad Software, San Diego, CA, USA) was used to analyze biochemical data (*n* = 5), and the results were presented as means ± SD. Additionally, one-way analysis of variance was used to investigate significant differences among distinct groups. SIMCA (V16.0.2, Sartorius Stedim Data Analytics AB, Umea, Sweden) was employed to conduct orthogonal partial least-squares discriminant analysis (OPLS-DA) and principal component analysis (PCA). Volcano plots were processed by R (V3.3.5), while Venn plots were processed using R (V1.6.20, 1.4.0). Heatmaps were generated using R (V1.0.12). Pathway analysis was conducted using R (V1.46.0, 3.3.5). Significant differentiation of metabolites among distinct groups was assessed by the unpaired two-tailed student’s *t*-test. Spearman correlation analysis was performed using R (V4.0.0).

## 3. Results

This section may be divided into subsections. It will provide a concise and precise description of the experimental results, their interpretation, as well as the experimental conclusions that can be drawn.

### 3.1. The Impact of AX on Liver Functional Indicators

**Figure 2:**
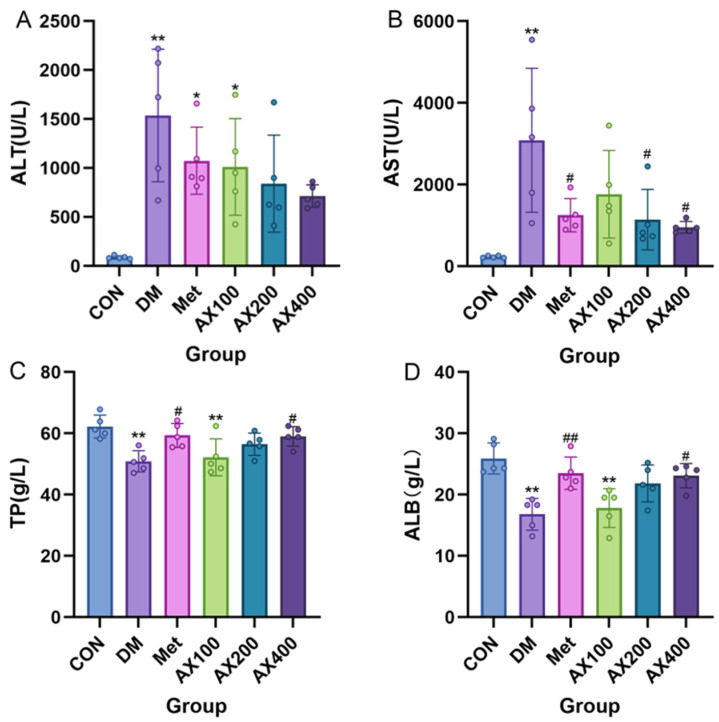
The effect of AX on liver functional indicators in type 2 diabetic mice. The effect of AX on (**A**) alanine aminotransferase (ALT), (**B**) aspartate aminotransferase (AST), (**C**) total protein (TP), (**D**) albumin (ALB). One-way analysis of variance (ANOVA) with Tukey’s post hoc test is used for intergroup comparisons. * *p* < 0.05, ** *p* < 0.01 comparison with CON group; # *p* < 0.05, ## *p* < 0.01 for comparison with the DM group. CON: control, DM: diabetes mellitus, Met: metformin, AX100: low-dose AX, AX200: medium-dose AX, and AX400: high-dose AX.

The results of the four liver function indicators are shown in Figure 2. Compared to the CON group, mice with type 2 diabetes showed a significant increase in serum levels of ALT and AST, while TP and ALB levels exhibited a significant decrease. After four weeks of treatment, both moderate and high doses of AX improved serum ALT, but the effect was not significant (Figure 2A). Compared with the DM group, AX400 significantly improved serum AST, TP, and ALB levels after four weeks of treatment (Figure 2B–D), and AX200 significantly improved serum AST levels (Figure 2B).

These results suggested that severe liver damage occurred in type 2 diabetic mice’s livers. AX exhibited a specific hepatoprotective effect, among which AX at 400 mg/kg BW is the most obvious.

### 3.2. The Effect of AX on Hepatic Tissue

**Figure 3:**
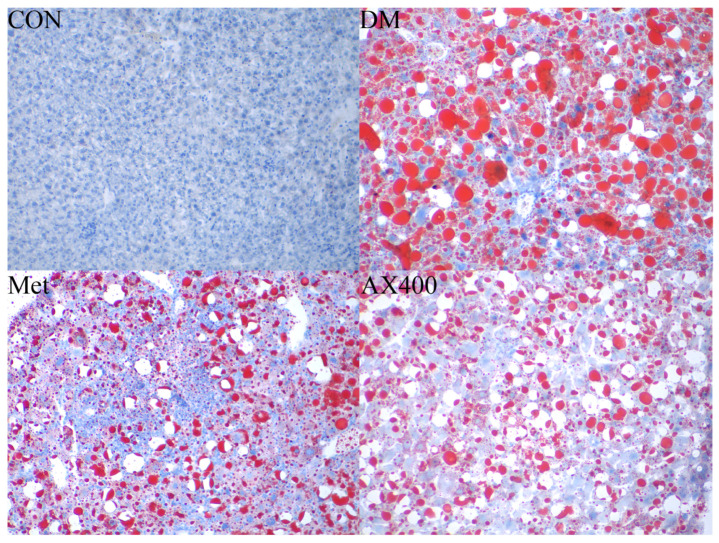
The effect of AX on hepatic pathological changes. Liver histological observation of the oil red O sections (original magnification ×400) of mice in CON, DM, Met, and AX400 groups. CON: control, DM: diabetes mellitus, Met: metformin, and AX400: high-dose AX.

In Figure 3, the oil red O staining of liver tissue in each group of mice is presented. The results showed that, compared to the CON group, abnormally large red lipid droplets were observed in the liver of the DM group. However, in the Met and AX400 groups, it was evident that the red lipid droplets became smaller in size and decreased in number. This indicated that both Met and AX400 had the ability to mitigate hepatic lipid accumulation caused by type 2 diabetes.

### 3.3. The Effect of AX on Hepatic Metabolism

#### 3.3.1. Multivariate Statistical Analysis

**Figure 4:**
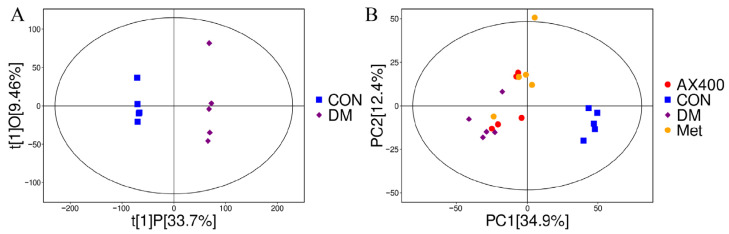
Effect of AX400 on hepatic metabolism in type 2 diabetic mice. (**A**) OPLS-DA score plots between DM and CON groups in the positive pattern, (**B**) PCA score plots among DM, CON, Met, and AX400 groups. CON: control, DM: diabetes mellitus, Met: metformin, and AX400: high-dose AX.

The study mentioned previously indicated that AX had a pronounced beneficial impact on both liver function indicators and the accumulation of lipids. Moreover, we investigated the regulatory effects of AX400 on hepatic metabolism in mice with type 2 diabetes. The OPLS-DA method was employed to analyze the metabolomics data of samples from the DM, CON, Met, and AX400 groups. As demonstrated by the complete separation of the CON and DM groups in the OPLS-DA score plot (Figure 4A), it could be concluded that significant changes to the endogenous metabolites of the mice had occurred after the model was established.

PCA was employed to better reveal the differences among samples belonging to different groups. The proximity of dots on the diagram reflects the degree of similarity in metabolite expression patterns among samples, with closer dots indicating a greater resemblance. As we can see from the PCA score plots, PC1 and PC2 of metabolomics were 34.9% and 12.4%, respectively, and a separation trend can be observed between the AX400 group and the DM group (Figure 4B), which indicates the regulatory impact of AX400 on hepatic metabolism.

#### 3.3.2. Screen and Identification of Potential Biomarkers

**Figure 5:**
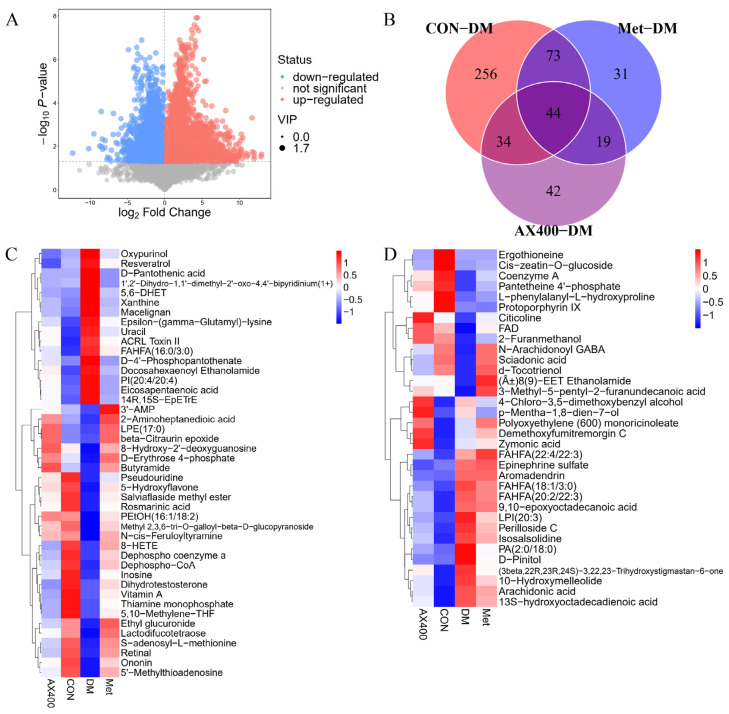
(**A**) Volcano plot for group CON vs DM. (**B**) Venn plot among CON, Met, AX400 groups compared to DM (*p* < 0.05, VIP > 1). (**C**) Heatmaps of differential metabolites in part 44. (**D**) Heatmaps of differential metabolites in part 34; rows: metabolites; columns: groups. CON: control, DM: diabetes mellitus, Met: metformin, and AX400: high-dose AX.

In this study, VIP > 1 and *p* < 0.05 were used as the criteria to screen important differential metabolites, and we identified 407 differential metabolites between the CON group and the DM group (Supplementary Table S2). The screening results of CON-DM were presented in the form of a volcano plot visualization (Figure 5A), which could visually show the overall distribution of metabolite differences. Likewise, a total of 167 distinct metabolites were discerned between the Met group and the DM group (Supplementary Table S3), while 139 differential metabolites were found to be distinct between the AX400 group and the DM group (Supplementary Table S4). The Venn (Figure 5B) diagram was used to show the relationship between the differential metabolites of each comparison group. Both AX400 and Met significantly regulated 44 metabolites, while AX400 alone regulated 34 metabolites, indicating the protective effects of both compounds against T2DM in mice.

Furthermore, according to the abundance value of every differential metabolite in parts 44 and 34, heatmaps (Figure 5C,D) were generated to directly visualize the inter-group relationships. Abnormal levels of differential metabolites were observed in both the 44 and 34 fractions of the DM group relative to those of the CON group. AX400 and Met were able to provide beneficial improvements in 44 metabolites (28 upregulated and 16 downregulated), and AX400 was able to improve 27 of 34 (12 upregulated and 15 downregulated).

### 3.4. Metabolic Pathway Analysis

**Figure 6:**
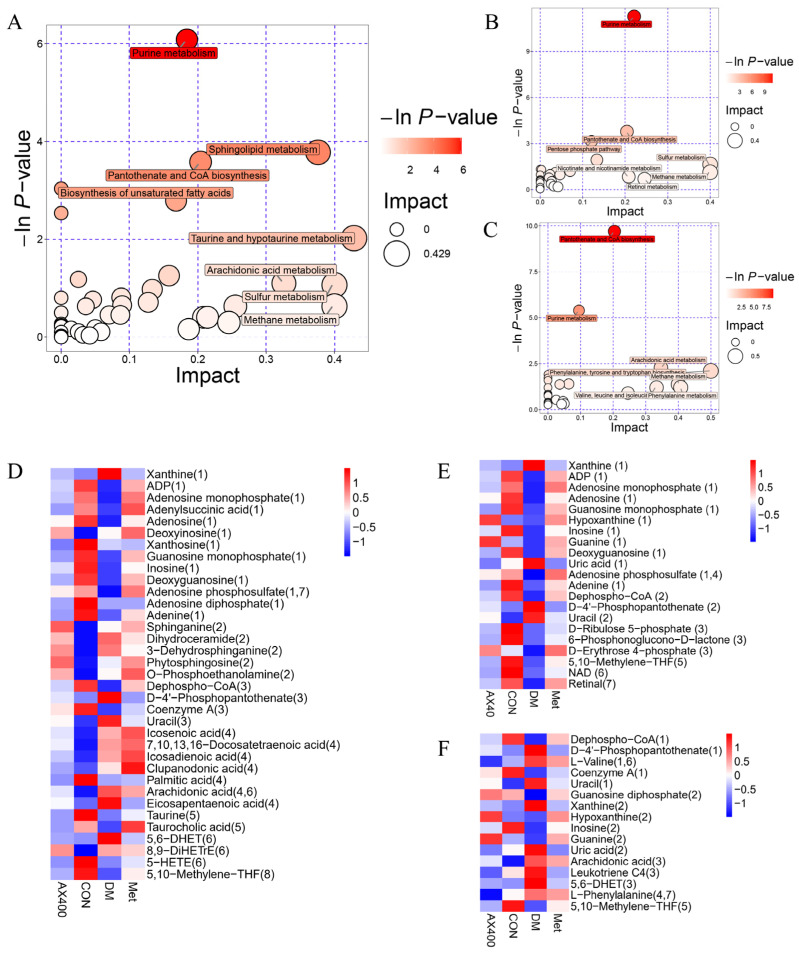
Summary plots for pathway analysis of (**A**) CON vs DM, (**B**) Met vs DM, (**C**) AX400 vs DM. Heatmaps of the abundance of metabolites involved in differential metabolic pathways in each comparison group: (**D**) CON vs DM, (**E**) Met vs DM, (**F**) AX400 vs DM. CON: control, DM: diabetes mellitus, Met: metformin, and AX400: high-dose AX.

The hepatic metabolites with significant changes in mice were subjected to metabolic pathway enrichment analysis (Figure 6A–C). We identified 14 pathways (*p* < 0.05 or impact value > 0.1) that may be perturbed in type 2 diabetic mice, and out of these, seven were found to be regulated by AX400, which contained (1) pantothenate and CoA biosynthesis, (2) purine metabolism, (3) arachidonic acid metabolism, (4) phenylalanine, tyrosine and tryptophan biosynthesis, (5) methane metabolism, (6) valine, leucine and isoleucine biosynthesis, and (7) phenylalanine metabolism. Pantothenate and CoA biosynthesis, as well as purine metabolism, were the two pathways that exhibited the most significant regulation by AX400.

According to the metabolic pathways, heatmaps were constructed to display the abundances of metabolites involved in these pathways (Figure 6D–F). Figure 6F showed that AX400 affected the seven metabolic pathways by regulating the levels of 16 differential metabolites (upregulate dephospho-CoA (1), coenzyme A (1), Guanosine diphosphate (2), hypoxanthine (2), inosine (2), uric acid (2), L-phenylalanine (4,7), and downregulate D-4′-phosphopantothenate (1), L-valine (1,6), uracil (1), xanthine (2), guanine (2), arachidonic acid (3), leukotriene C4 (3), 5,6-DHET (3), 5,10-methylene-THF (5)). The trend of perturbed metabolites in the callback indicated that AX-induced protection against T2DM in mice was associated with metabolic pathways.

### 3.5. Correlation Analysis

The aforementioned findings suggest that AX400 exerts a regulatory effect on perturbed differential metabolites in T2DM mice. To investigate the correlation between differential metabolites and diabetes phenotype, we conducted Spearman correlation analysis to examine their relationship with liver functional indicators (ALT, AST, TP, and ALB). Metabolites were derived from Venn diagrams 44 and 34, as well as from parts involved in metabolic pathways in the CON-DM comparison group. 

**Figure 7:**
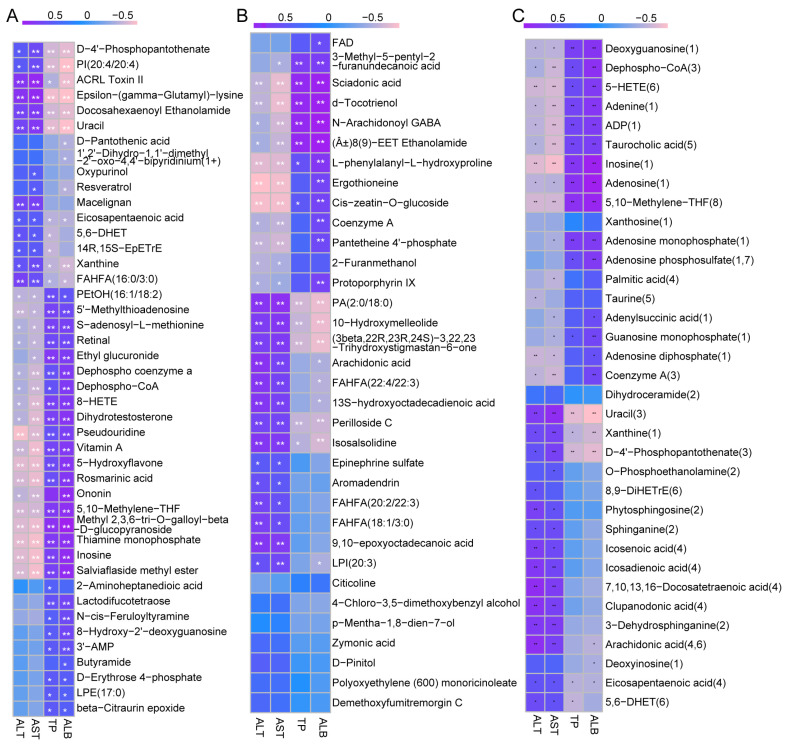
The Spearman correlation between different metabolites and 4 liver functional indicators of type 2 diabetic mice: (**A**) 44, (**B**) 34, and (**C**) metabolic pathways in CON-DM. The R values are represented by gradient colors, where purple and pink cells indicate positive and negative correlations, respectively; * *p* < 0.05, ** *p* < 0.01. ALT: alanine aminotransferase, AST: aspartate aminotransferase, TP: total protein, and ALB: albumin.

Twenty-seven out of forty-four differential metabolites and eleven out of thirty-four metabolites showed significant correlations with all four liver function indicators (Figure 7A,B). These 38 metabolites can be classified into amino acids/peptides, purines/pyrimidines/nucleotides, cholesterol/lipids, ketones/hydroxyl acids, vitamins/provitamins, and others by category. In addition, these metabolites accounted for a relatively large proportion of cholesterol/lipids and ketones/hydroxyl acids.

Figure 7C presented extremely high correlations between metabolites (deoxyguanosine, dephospho−CoA, 5−HETE, adenine, ADP, taurocholic acid, inosine, adenosine, 5,10−methylene−THF, uracil, xanthine, D−4′−phosphopantothenate) and the four liver functional indicators. According to the above results, D−4′−phosphopantothenate, uracil, dephospho−CoA, inosine, xanthine, 5,10−methylene−THF were involved in metabolic pathways (pantothenate and CoA biosynthesis, purine metabolism, and methane metabolism), further indicating that pantothenate and CoA biosynthesis, as well as purine metabolism, were the main metabolic pathways through which AX regulates hepatic metabolic disorders.

## 4. Discussion

In our present study, the AX extracted from wastewater produced during vital wheat gluten protein production was used for intervention in type 2 diabetic mice. The hepatoprotective impacts of AX on type 2 diabetic mice were evaluated through biochemical indicator determination, oil red O staining, and nontargeted metabolomics analysis. The results showed that AX positively regulated the levels of four liver function indicators in mice serum and improved hepatic lipid accumulation. The liver metabolomics results showed that endogenous metabolic disorders occurred in the liver of T2DM mice, and intergroup differences in metabolites involved multiple metabolic pathways. AX was observed to effectively improve the abundance of different metabolites, thereby regulating relevant metabolic pathways. In particular, two significant pathways (pantothenate and CoA biosynthesis, as well as purine metabolism) were found to be regulated by AX.

Compared with the CON group, the DM group of mice showed abnormal levels of ALT, AST, TP, and ALB in serum, indicating impaired liver function in the mice. After 4 weeks of AX intervention, compared with the DM group, high-dose AX significantly improved the levels of serum AST, TP, and ALB and also had a certain improvement effect on ALT levels. Similarly, studies showed that red kidney bean polysaccharide and small black soybean polysaccharide also had a positive improvement effect on these functional indicators, and the higher the purity of the polysaccharide, the more pronounced the effect [27]. Oil red O staining results showed a reduction in red lipid droplets on liver slices after four weeks of high-dose AX intervention. Similar results were obtained in the study of the effect of Ganoderma lucidum polysaccharides on diabetes [28]. These results demonstrate the potential benefits of dietary fibers, including AX, on diabetic liver disease, and that the purity of dietary fibers has a certain influence on their effectiveness.

Pantothenate and CoA biosynthesis play pivotal roles in multiple physiological and pathological processes within cells [29]. Pantothenic acid (PA) is a water-soluble vitamin that belongs to the B-complex group and is commonly referred to as vitamin B5. It is one of the components of coenzyme A (CoA), and therefore is also known as a precursor to CoA [30]. Supplementing rats’ diets with PA can result in an improvement of various symptoms associated with type 2 diabetes [29]. CoA is an important coenzyme that is widely involved in energy metabolism, helping to convert glucose, fats, and amino acids into energy [31]. PA can be converted into CoA after multiple steps, which is one of the necessary raw materials utilized in the synthesis of CoA. It is noteworthy that the DM group of mice exhibited a higher level of PA and a lower level of CoA in the liver compared to the CON group in the current study. Additionally, the content of pantetheine 4′-phosphate, which is an important intermediate product responsible for synthesizing CoA from PA, was found to be lower in the DM group, which could lead to the excessive accumulation of PA and another intermediate product (D-4′-phosphopantothenate). Interestingly, in the AX400 group, the content of PA and D-4′-phosphopantothenate was similar to that of the CON group, while the levels of pantetheine 4′-phosphate and CoA were reversed. The correlation analysis results suggest that CoA and pantetheine 4′-phosphate have a significant positive correlation with ALB, while demonstrating a noteworthy inverse correlation with AST and ALT. These findings suggest that the intermediate pathway responsible for synthesizing CoA from PA may be inhibited in the liver of mice with type 2 diabetes, resulting in a decrease in CoA levels and promoting metabolic disorders in the liver. AX400 has a positive regulatory effect on this phenomenon.

In purine metabolism, both the biosynthesis and catabolism of purine nucleotides are involved [32]. During purine degradation, purine nucleotides are converted into xanthine and guanine, which are then transformed into hypoxanthine through enzymes such as purine nucleotide phosphorylase. Finally, xanthine oxidase oxidizes xanthine to generate uric acid, which is the end product of purine metabolism [33]. Elevated serum uric acid levels have been associated with various health problems, such as hypertension, cardiovascular disease, chronic kidney disease, insulin resistance, and diabetic microvascular complications [34]. Purine metabolism mainly occurs in organs such as the liver, which plays an important role in maintaining uric acid levels in the body. Our study found that, compared to the CON group, the level of uric acid in the liver of type 2 diabetic mice was significantly elevated. This may be due to metabolic disorders in diabetic mice, leading to increased blood lipid levels, lipid peroxidation, and increased xanthine oxidase activity [34]. We also observed an increase in xanthine levels and a decrease in hypoxanthine levels, which may be caused by enhanced purine nucleotide phosphorylase activity, resulting in further oxidative stress damage to the body [35]. After AX intervention, we found that the levels of uric acid and xanthine in the liver of T2DM mice improved. This suggested that AX may have a regulatory effect on purine metabolism in the liver.

## 5. Conclusions

The results of this study showed that a high dose of AX extracted from wastewater produced during vital wheat gluten protein production had a significant improvement effect on certain liver function indicators and could effectively reduce liver lipid accumulation in type 2 diabetic mice based on the results of oil red O staining. In addition, high-dose AX regulated important different metabolites, affecting metabolic pathways, such as pantothenate and CoA biosynthesis, and purine metabolism, further improving the liver function of type 2 diabetic mice. In summary, this study elucidated, to some extent, the protective effects of AX on the liver of type 2 diabetic mice from the perspective of metabolomics, providing a reference for the development of new type 2 diabetic functional foods aimed at improving liver function. The limitation of this study is that the specific mechanisms of action of metabolic pathways and metabolites, as well as their association with liver function, remain unclear. In the future, it will be necessary to conduct further validation and research on relevant biological processes and further consider their effects in real-life human environments.

## Data Availability

The data presented in this study are available upon request from the corresponding author.

## Supplementary Materials

The following supporting information can be downloaded at: https://www.mdpi.com/article/10.3390/foods12142640/s1, Table S1: Molecular weights and monosaccharide composition of AX; Table S2: 407 vital metabolites in the CON group compared with the DM group in hepatic metabolism; Table S3: 167 vital metabolites in the Met group compared with the DM group in hepatic metabolism; Table S4: 139 vital metabolites in the AX400 group compared with the DM group in hepatic metabolism.
