# Supplementary material for: Effect of Arabinoxylan from Wastewater Generated during Vital Wheat Gluten Production on Liver Metabolism in Type 2 Diabetic Mice
Source: Foods. 2023 Jul 8;12(14):2640. doi: 10.3390/foods12142640 (PMC10378226; doi:10.3390/foods12142640)
Supplement: Supplementary file 1 [file foods-12-02640-s001.zip › foods-2446613-supplementary.pdf]

Table S1. Molecular weights and monosaccharide composition of AX.

Polysaccharides	AX/%	Molecular Weights	Monosaccharide Compositions
AX	97.62	45745kDa	Ara: Xyl: Glc: Gal: Man: GalA=39.88:37.92:9.48:7.97:4.08: 0.67

Ara, arabinose; Xyl, xylose; Glc, glucose; Gal, galatose; Man, mannose; GalA, galacturonic acid.

Table S2. 407 vital metabolites in CON group compared with DM group in hepatic metabolism.

**Metabolites**	***p*-value**	**Fold Change**	**VIP**
Adenosine	0.0053	4.5860	1.5705
Adenosine monophosphate (AMP)	0.0150	11.0901	1.6126
1-Butylamine	0.0096	0.9314	1.3124
Aminoacetone	0.0362	0.9398	1.1613
Glycohyocholic acid	0.0019	9.5737	1.6594
Taurine	0.0065	1.6353	1.3779
sn-Glycerol3-phosphate	0.0009	13.2596	1.5647
Guanosine-5'-monophosphate	0.0001	6.9344	1.5811
Adenosine 5'-diphosphate	0.0005	2.5986	1.5489
Nicotinamide	0.0001	1.4748	1.6194
Xanthosine	0.0095	2.4149	1.2058
Glycerophosphocholine	0.0006	3.1948	1.4160
L-Pipecolic acid	0.0000	7.4181	1.1501
D-Pantothenic acid	0.0000	0.4420	1.5736
Dephospho coenzyme a	0.0058	4.8586	1.6327
(9S,10E,12Z,15Z)-9-Hydroxy-10,12,15-octadecatrienoic acid	0.0202	0.2116	1.5438
Inosine	0.0000	6.5387	1.6792
Guanosine monophosphate	0.0006	8.8482	1.6004
ADP	0.0008	2.2360	1.5141
L-Malic acid	0.0046	1.1493	1.3972
L-Malic acid	0.0008	1.7072	1.5179
Liquiritigenin 4'-[3-acetylapiosyl-(1->2)-glucoside]	0.0466	6.3861	1.4181
Xanthine	0.0008	0.4962	1.5527
L-Ascorbic acid	0.0354	1.5260	1.0575
3-Phosphoglyceric acid	0.0065	0.3330	1.3710
Uridine 5'-monophosphate	0.0005	18.4825	1.6461
1-Methyl-L-Histidine	0.0410	3.2775	1.3576
Fumaric acid	0.0055	1.3484	1.3527
Sphinganine	0.0002	0.5410	1.5968
Biliverdin	0.0092	0.5173	1.5251
Deoxyinosine	0.0181	0.4004	1.2613
4-Dodecylbenzenesulfonic Acid	0.0074	1.5120	1.1983
Glucosamine 6-phosphate	0.0051	2.6296	1.4201
Adenosine diphosphate ribose	0.0138	93.9015	1.6492
3-Dehydrosphinganine	0.0045	0.3903	1.6096
Beta-Guanidinopropionic acid	0.0398	0.7587	1.1372
D-Glucose 6-Phosphate	0.0453	1.2620	1.0956
Malic acid	0.0087	1.2974	1.3046
Syringol	0.0007	0.8954	1.5007
Isoleucyl-Leucine	0.0464	2.0266	1.1861

Maltotetraose	0.0321	10.3547	1.5228
S-Adenosyl-L-homocysteine	0.0175	2.6152	1.4628
D(+)-Glucose	0.0019	2.1221	1.4765
Phaseollidin	0.0054	1.3818	1.3718
L-Lactic acid	0.0115	1.5815	1.3228
FAHFA(20:1/20:0)	0.0146	0.2431	1.3729
FAHFA(18:1/18:0)	0.0008	0.3269	1.5717
Protoanemonin	0.0015	2.3916	1.5067
LPG(18:0)	0.0248	0.3775	1.0805
Lactose	0.0113	5.4242	1.4800
FAHFA(22:4/22:3)	0.0004	0.2512	1.5270
L-Threonine	0.0016	0.5689	1.4678
Hexadecanedioic acid	0.0248	0.5998	1.1506
Leucyl-phenylalanine	0.0062	2.2873	1.3989
FAHFA(20:1/22:3)	0.0280	0.5120	1.2434
Kojibiose	0.0135	5.0001	1.4359
Acetylphosphate	0.0371	0.4237	1.1615
FAHFA(18:1/22:3)	0.0069	0.6479	1.3594
5-Hydroxyhexanoic acid	0.0206	0.2082	1.0511
Isoleucyl-Isoleucine	0.0064	4.0008	1.6068
FAD	0.0327	1.1669	1.1231
Galactinol	0.0098	8.7695	1.4672
Ergothioneine	0.0007	25.5138	1.7109
LPG(20:4)	0.0012	0.4513	1.5797
Oxypurinol	0.0185	0.6991	1.2378
Ononin	0.0007	5.0538	1.4022
Prolyl-Methionine	0.0152	3.1041	1.1824
FAHFA(20:2/22:3)	0.0053	0.3938	1.2106
5'-Methylthioadenosine	0.0028	4.6667	1.5702
D-Erythrose 4-phosphate	0.0012	5.9621	1.2388
LPE(18:0)	0.0009	0.4863	1.5029
Isobutyrylglycine	0.0025	3.1289	1.6188
LPE(17:0)	0.0486	6.5544	1.4454
Cholic acid	0.0207	8.9806	1.4273
Adenylsuccinic acid	0.0317	3.3661	1.2902
4-Hydroxy-2-butenoic acid gamma-lactone	0.0031	2.5142	1.5353
Salviaflaside methyl ester	0.0000	6.5804	1.6340
Hexylamine	0.0283	0.9113	1.1883
FAHFA(22:6/22:3)	0.0461	0.2053	1.2661
Dephospho-CoA	0.0039	4.2684	1.6321
PG(20:3/22:4)	0.0294	0.4560	1.1412
8-Hydroxy-2'-deoxyguanosine	0.0122	2.4641	1.2974
3'-AMP	0.0467	1.6826	1.1143
D-Ribulose 5-phosphate	0.0000	4.1590	1.6311

BMP(18:1/20:3)	0.0350	0.1178	1.5597
Adenosine monophosphate	0.0007	2.5574	1.4946
Butyrylcarnitine	0.0390	1.8661	1.1057
LPE(16:1)	0.0305	2.5495	1.3601
D-Sedoheptulose 7-phosphate	0.0009	2.9059	1.2371
Docosahexaenoyl Ethanolamide	0.0004	0.2920	1.5940
Thiamine monophosphate	0.0000	5.9538	1.6885
D-Xylose	0.0058	1.7363	1.3780
S-adenosyl-L-methionine	0.0072	5.5534	1.5271
Adenine	0.0076	2.9924	1.6191
PG(18:1/20:4)	0.0204	0.1946	1.6021
Arachidonic acid	0.0006	0.5460	1.5789
Docosapentaenoic acid (22n-3)	0.0025	0.3809	1.4304
BMP(18:1/22:4)	0.0275	0.2706	1.2523
Stearoylethanolamide	0.0007	0.4809	1.5007
Dehydroascorbic acid	0.0027	1.3672	1.4613
BMP(18:2/20:3)	0.0340	0.1653	1.5502
Hydroxyprolyl-Leucine	0.0040	2.4833	1.3460
PE(16:1e/20:4)	0.0217	0.6761	1.1681
PS(16:2/24:4)	0.0415	0.5063	1.3123
PG(18:1/20:2)	0.0257	0.0764	1.1617
PG(18:2/22:5)	0.0316	0.4764	1.3410
L-Gulonic gamma-lactone	0.0003	1.8072	1.4947
Epsilon-(gamma-Glutamyl)-lysine	0.0106	0.2088	1.6284
LysoPC(22:5(4Z,7Z,10Z,13Z,16Z))	0.0123	1.3665	1.2875
D-4'-Phosphopantothenate	0.0030	0.2007	1.6124
6-Phosphogluconic acid	0.0007	2.8332	1.4880
4-Chloro-3,5-dimethoxybenzyl alcohol	0.0174	0.8624	1.3153
Graveoline	0.0067	2.3372	1.1863
7-Ketodeoxycholic acid	0.0393	166.9213	1.5163
LPC(17:1)	0.0381	2.5123	1.3839
PG(20:4/22:5)	0.0187	0.2560	1.4995
(9xi,10xi,12xi)-9,10-Dihydroxy-12-octadecenoic acid	0.0047	0.4901	1.3763
Isoleucyl-Methionine	0.0136	2.7906	1.2459
Raffinose	0.0201	12.1627	1.5407
5-Methylcytidine	0.0201	7.7518	1.5702
Phytosphingosine	0.0066	0.5472	1.3673
H-PHE-PRO-OH	0.0095	2.3983	1.4822
PG(22:4/22:5)	0.0160	0.3767	1.4837
MG(18:2(9Z,12Z)/0:0/0:0)	0.0242	0.2331	1.5145
Retinal	0.0009	2.5950	1.6500
8-HETE	0.0000	3.3620	1.6651
Dihydrotestosterone	0.0012	3.9085	1.6481
Prostaglandin E2	0.0382	0.3333	1.1258

Maleamic acid	0.0021	1.5079	1.4628
3'-O-Methyl-D-adenosine	0.0249	2.3986	1.3606
BMP(20:4/22:6)	0.0160	1.3546	1.2493
Palmitoleoyl Ethanolamide	0.0130	0.3387	1.5627
ACar(20:3)	0.0294	0.4059	1.1551
16(17)-EpDPE	0.0089	0.2733	1.4872
Pseudouridine	0.0095	1.8468	1.3706
Folinic acid	0.0051	3.3111	1.5445
2-Furanmethanol	0.0148	1.7136	1.3017
Aspartame	0.0078	0.5488	1.3831
BMP(20:3/20:4)	0.0065	0.3069	1.4204
ACar(16:4)	0.0206	8.8553	1.3280
2-(3-Phenylpropyl)pyridine	0.0064	0.8722	1.3439
Lysyl-Leucine	0.0469	1.8171	1.0581
Androstanediol	0.0000	4.2827	1.6375
p-Cresol glucuronide	0.0281	2.5152	1.1962
Hinokitiol;beta-Thujaplicin	0.0049	0.2391	1.6021
9,10-epoxyoctadecanoic acid	0.0051	0.2333	1.6195
Phenylalanyl-Valine	0.0135	2.0739	1.2871
beta-D-Glucosamine	0.0421	1.6374	1.1852
Valyl-Arginine	0.0320	3.5406	1.4812
Glutaminyltyrosine	0.0178	1.6507	1.1900
(3R, 6'Z)-3,4-Dihydro-8-hydroxy-3-(6-pentadecenyl)-1H-2-benzopyran-1-one	0.0032	15.9467	1.5251
PI(20:3(5Z,8Z,11Z)/16:0)	0.0034	0.2451	1.2726
2,4,5,7alpha-Tetrahydro-1,4,4,7a-tetramethyl-1H-inden-2-ol	0.0315	1.3526	1.2153
Lysyl-Hydroxyproline	0.0174	1.6422	1.2197
Methionyl-Alanine	0.0045	0.6429	1.3877
PC(2:0/16:2)	0.0091	0.3057	1.5149
L-Erythrulose	0.0015	1.9275	1.4819
ACRL Toxin II	0.0018	0.0372	1.4894
Maltohexaose	0.0189	7.8123	1.5445
Stearoylglycine	0.0012	0.0786	1.6191
OxPG(18:1/18:1+3O)	0.0171	0.1542	1.5797
3-Indoleacrylic acid	0.0431	0.7984	1.1357
Trigonelline	0.0001	3.2699	1.6427
Glucosylisomaltol	0.0145	6.0834	1.3770
BMP(22:4/22:6)	0.0011	0.5418	1.4039
PEtOH(16:1/18:2)	0.0469	9.5960	1.5526
PC(2:0/20:2)	0.0220	1.8206	1.2530
p-Mentha-1,8-dien-7-ol	0.0456	0.9300	1.0978
FAHFA(20:2/18:0)	0.0091	0.1920	1.5445
β-Alanine	0.0329	0.6743	1.3143
Amylopectin	0.0200	8.9439	1.4011
29-Norcycloartane-3,24-dione	0.0233	0.1075	1.4539

Hyperoside	0.0442	0.3955	1.0794
FAHFA(18:1/3:0)	0.0211	0.2156	1.4218
CPA(18:0/0:0)	0.0013	0.3303	1.3611
2-(3-Phenylpropyl)tetrahydrofuran	0.0435	0.8216	1.0976
Diosmetin	0.0270	1.9614	1.0623
Methyl 2-(10-heptadecenyl)-6-hydroxybenzoate	0.0221	0.2740	1.4473
Sciadonic acid	0.0027	73.5528	1.6689
5-Aminopentanal	0.0153	0.6963	1.3007
PG(18:1/22:4)	0.0195	0.1992	1.5140
8,9-DiHETrE	0.0000	0.2786	1.4898
Hydrocortisone	0.0088	0.1870	1.4631
Trehalose	0.0032	3.6062	1.4363
2-Furoic acid	0.0199	1.9686	1.3154
4,8,12-Trimethyltridecanoyl-CoA	0.0297	0.4474	1.1365
2-Aminoheptanedioic acid	0.0298	2.5413	1.2285
Maltopentaose	0.0254	6.0121	1.4684
N-Arachidonoyl GABA	0.0019	17.5236	1.5674
Troxilin B3	0.0047	29.0071	1.6878
trans-Octadec-2-enoyl-CoA	0.0021	0.5828	1.4399
Uracil	0.0000	0.2002	1.5639
LPS(20:1)	0.0344	2.2704	1.0040
O-Phosphoethanolamine	0.0262	0.6175	1.2019
16-Hydroxy hexadecanoic acid	0.0427	0.7047	1.1530
PG(18:2/22:4)	0.0027	0.3230	1.4881
6-Phosphonoglucono-D-lactone	0.0003	3.6077	1.5891
Tyrosyl-Proline	0.0069	1.7832	1.3732
Polyoxyethylene (600) monoricinoleate	0.0038	0.5542	1.4213
LysoPC(17:0)	0.0086	0.4597	1.3105
PG(20:4/22:4)	0.0008	0.3429	1.5711
Glycitin	0.0186	5.0229	1.4768
Coenzyme A	0.0009	4.0107	1.4740
PG(16:0/20:4)	0.0265	0.3506	1.3521
PC(12:0/22:2)	0.0476	0.5595	1.2330
Ethyl glucuronide	0.0074	6.1315	1.6063
indolin-2-one	0.0254	1.7748	1.1985
PG(18:0/20:4)	0.0088	0.3474	1.5615
(Â±)8(9)-EET Ethanolamide	0.0048	8.4402	1.5375
Dihydro-3-(1-octenyl)-2,5-furandione	0.0000	4.2171	1.5723
L-Alanyl-L-Glutamine	0.0482	4.9293	1.1259
5-HETE	0.0058	4.0609	1.6399
Abscisic acid	0.0012	0.8785	1.4866
FAHFA(16:0/3:0)	0.0002	0.3530	1.4559
N-Hexadecanoylpyrrolidine	0.0006	0.4885	1.5061
FAHFA(16:0/22:3)	0.0142	0.6983	1.3321

Pyridoxal 5'-phosphate	0.0198	4.3057	1.4049
Hexadecanedioic acid mono-L-carnitine ester	0.0309	14.5315	1.5469
15-HETE	0.0271	0.2914	1.4372
BMP(20:3/22:5)	0.0091	0.2736	1.5359
BMP(20:3/22:6)	0.0057	0.2805	1.4682
Deoxyguanosine	0.0015	5.1921	1.5231
LPS(18:0)	0.0088	0.7461	1.3512
PG(18:1/22:6)	0.0045	0.3227	1.4726
Lactodifucotetraose	0.0085	1.9567	1.3784
LysoPC(O-18:0)	0.0004	0.3592	1.5968
(8E,15E)-1,8,15-Heptadecatriene-11,13-diyne	0.0374	0.8080	1.1488
1-Oleoylglycerophosphoinositol	0.0387	0.7459	1.1085
7alpha-Hydroxy-3-oxo-5beta-cholan-24-oic acid	0.0286	6.8821	1.2618
PG(18:1/20:3)	0.0205	0.1980	1.5999
BMP(18:2/22:4)	0.0136	0.2933	1.2720
3-Methyl-5-pentyl-2-furanundecanoic acid	0.0194	8.4838	1.2626
Eicosadienoic acid	0.0047	0.4966	1.3160
PA(2:0/18:0)	0.0307	0.0580	1.5243
7-Aminomethyl-7-carbaguanine	0.0371	2.0089	1.3199
PC(14:1/24:4)	0.0080	0.7197	1.3167
Biochanin A	0.0225	68.2332	1.6251
PI(18:1/20:4)	0.0046	0.5750	1.2522
PI(18:0/20:4)	0.0103	0.6276	1.5059
5-Hydroxyflavone	0.0000	11.6555	1.4534
beta-D-Galactopyranosyl-(1->2)-[beta-D-galactopyranosyl-(1->4)]-D-galactose	0.0198	12.2566	1.5439
alpha-Terpinyl anthranilate	0.0120	2.2958	1.2537
PI(16:1/20:4)	0.0214	1.5867	1.2021
3-Hydroxychavicol 1-[rhamnosyl-(1->6)-glucoside]	0.0132	3.2353	1.4006
PG(16:0/22:6)	0.0200	0.3294	1.4488
15-Deoxy-d-12,14-PGJ2	0.0216	0.2710	1.3053
gamma-Aminobutyric acid	0.0056	0.3186	1.2886
H-GLU(ALA-OH)-OH	0.0208	1.8054	1.2002
13S-hydroxyoctadecadienoic acid	0.0002	0.2562	1.5542
4-(Trimethylammonio)butanoate	0.0037	4.5640	1.6471
16-Methylheptadecanoic acid	0.0000	0.5928	1.6486
4-Aminoantipyrine	0.0242	0.8985	1.1736
5,10-Methylene-THF	0.0263	14.4678	1.5593
NAD	0.0250	1.8702	1.4081
7a,12a-Dihydroxy-5a-cholestan-3-one	0.0015	0.4128	1.4140
Alanyl-Leucine	0.0017	2.1294	1.5099
6,7,4'-Trihydroxyisoflavone	0.0347	528.8381	1.6310
PE(18:1e/20:4)	0.0081	0.3430	1.2599
Perilloside C	0.0034	0.2610	1.2377
trans-Zeatin-riboside	0.0004	3.1508	1.5293

Norchalciporyl propionate	0.0377	0.2451	1.1348
Traumatin	0.0000	2.5232	1.6639
Taurocholic acid	0.0210	2.2173	1.2182
Tetradecanedioic acid	0.0217	0.5291	1.2261
D-Pinitol	0.0024	0.4949	1.4572
11Z-Eicosenoic acid	0.0046	0.4855	1.3755
PI(20:4/20:4)	0.0001	0.2747	1.6322
3-beta-Glucosylcellotriose	0.0296	7.9135	1.4941
LPI(18:0)	0.0001	0.5266	1.5401
LPS(22:2)	0.0180	0.2906	1.5016
PG(16:3/22:6)	0.0413	0.3772	1.4091
Isosalsolidine	0.0051	0.0681	1.2371
PC(14:0e/4:0)	0.0244	0.6844	1.2177
1'-O-Acetylpaxilline	0.0002	13.5743	1.6505
Mangiferdesmethylursanone	0.0016	0.2244	1.4445
Tryptophyl-Asparagine	0.0256	2.3058	1.1830
PI(18:1/18:1)	0.0119	0.6160	1.2916
Cytochalasin Ppho	0.0338	2.1670	1.1746
LPI(20:3)	0.0264	0.4722	1.1792
Indoxyl sulfate	0.0025	2.4377	1.4016
5-Hydroxythalidomide	0.0018	6.9142	1.6130
Pantetheine 4'-phosphate	0.0103	3.1573	1.5829
Adenosine phosphosulfate	0.0024	1.6999	1.4465
Vanilloloside	0.0150	2.1315	1.3343
L-2-Amino-5-hydroxypentanoic acid	0.0302	1.6710	1.0708
Dihydroceramide	0.0429	0.9184	1.1024
beta-Citraurin epoxide	0.0423	7.0273	1.5249
Monomethyl glutaric acid	0.0368	2.2751	1.2886
Tyrosyl-Tryptophan	0.0316	1.7282	1.0992
L-phenylalanyl-L-hydroxyproline	0.0042	5.6128	1.6373
Palmitic acid	0.0014	1.9062	1.5334
Maslinic acid	0.0469	736.7340	1.7001
Ercalcitriol	0.0142	0.2505	1.5097
Diethylphosphate	0.0137	0.8569	1.2682
11,14,17-Eicosatrienoic acid	0.0016	0.3084	1.4988
Agrocybenine	0.0334	0.8951	1.1457
Pentosidine	0.0142	1.7146	1.2800
LysoPA(18:1(9Z)/0:0)	0.0024	0.5181	1.4222
Acetylleucine	0.0314	2.2984	1.3091
Oxoadipic acid	0.0006	2.1758	1.3851
Uridine 5'-diphosphoglucuronic acid (UDP-D-glucuronate)	0.0467	3.6267	1.3830
Ginkgolide A	0.0007	0.3452	1.0091
Chrysin	0.0414	72.0146	1.4830
Lansiumamide A	0.0060	113.7913	1.6266

PS(20:4(5Z,8Z,11Z,14Z)/22:2(13Z,16Z))	0.0135	0.2320	1.5451
Isovalerylglutamic acid	0.0195	0.9000	1.2953
Mutatoxanthin	0.0443	0.4596	1.2579
Maltotriose	0.0144	10.3445	1.5198
Isoliquiritigenin	0.0225	0.4561	1.4627
Carnosic acid	0.0012	2.4733	1.5218
(3beta,23E)-3-Hydroxy-27-norcycloart-23-en-25-one	0.0000	0.2418	1.5312
Fenugreekine	0.0207	1.4377	1.2300
3-Hydroxysebacic acid	0.0205	0.3264	1.2793
PI(18:1(9Z)/18:1(9Z))	0.0106	0.2421	1.5320
5,6-DHET	0.0014	0.5052	1.4177
Pyridine	0.0027	1.2992	1.4379
Butyramide	0.0174	1.2007	1.2079
Lysyl-Glycine	0.0014	8.0989	1.6465
Sissotrin	0.0113	5.8647	1.4626
Adrenic acid	0.0002	0.4422	1.5127
13-OxoODE	0.0149	0.1210	1.6549
Cis-zeatin-O-glucoside	0.0051	51.5360	1.6964
S-(Formylmethyl)glutathione	0.0026	8.7268	1.6546
PG(18:1(11Z)/20:3(5Z,8Z,11Z))	0.0438	0.0247	1.4493
Umbelliprenin	0.0438	0.4335	1.1857
Dihydrojasmonic acid	0.0196	1.0842	1.2499
(5a,6a,8a,11a)-8-Hydroxy-2-oxo-1(10),3-guaiadien-12,6-olide-15-al 8-(4-hydroxyphenylacetate)	0.0009	0.4241	1.4271
10-Hydroxymelleolide	0.0045	0.1881	1.6603
Schidigeragenin C	0.0116	2.4725	1.2525
Alline	0.0091	0.8596	1.3020
(6b,7b,13R)-6,7-Diacetoxy-8,14-labdadiene-13-ol	0.0264	6.9469	1.4036
PGD2 ethanolamide	0.0189	0.3381	1.4883
Phenylalanyl-Lysine	0.0484	2.5028	1.2339
Rosmarinic acid	0.0000	5.5625	1.6153
4-Methoxy-5-(3,7,11,15-tetramethyl-2,6,10,14-hexadecatetraenyl)-1,3-benzenediol	0.0109	14.6125	1.6156
2',7-Dihydroxy-4'-methoxy-8-prenylflavan 2',7-diglucoside	0.0128	0.7313	1.2727
Eicosapentaenoic acid	0.0010	0.5217	1.5283
Vitamin A	0.0000	4.7079	1.6619
Diethylhexyl adipate	0.0317	1.3782	1.0889
CPA(18:1(9Z)/0:0)	0.0301	0.7214	1.1336
Inositol cyclic phosphate	0.0182	2.3693	1.4280
PS(20:3(8Z,11Z,14Z)/22:2(13Z,16Z))	0.0090	0.2068	1.3273
14R,15S-EpETrE	0.0001	0.3863	1.4371
PG(18:1(11Z)/22:5(4Z,7Z,10Z,13Z,16Z))	0.0241	0.2031	1.4286
2-Methyl-1,3-cyclohexadiene	0.0377	0.7958	1.1283
(3beta,22R,23R,24S)-3,22,23-Trihydroxystigmastan-6-one	0.0000	0.2068	1.5987
2,6-Dimethoxy-4-propylphenol	0.0005	3.6375	1.4768
Methyl 2,3,6-tri-O-galloyl-beta-D-glucopyranoside	0.0000	3.3817	1.5426

1,1'-[1,12-Dodecanediylbis(oxy)]bisbenzene	0.0217	7.7387	1.3824
5-Methyl-2,4-bis(3-methyl-2-butenyl)-6-(2-methyl-1-oxopropyl)-5-(4-methyl-3-pentenyl)cyclohexanone	0.0278	0.2019	1.4129
Tyrosyl-Histidine	0.0183	3.3919	1.4193
Prolyl-Valine	0.0276	2.0812	1.1052
ADP-ribose 2'-phosphate	0.0037	2.4009	1.3503
6-(alpha-D-Glucosaminyl)-1D-myo-inositol	0.0152	3.9270	1.4690
3'-Sialyllactose	0.0304	5.0322	1.3778
Clupanodonyl carnitine	0.0132	0.3135	1.2409
Fragransin B2	0.0056	8.1260	1.4432
Suberylglycine	0.0043	71.6142	1.7085
Progesterone	0.0223	4.3075	1.5182
Polysorbate 60	0.0001	0.2456	1.4464
N-cis-Feruloyltyramine	0.0005	2.3762	1.5270
Demethoxyfumitremorgin C	0.0205	0.0002	1.6913
Mizolastine	0.0000	0.7923	1.6703
Tyrosyl-Alanine	0.0187	3.1771	1.2894
Gyrocyanin	0.0354	0.7228	1.1751
PI(20:3(5Z,8Z,11Z)/18:0)	0.0001	0.6212	1.5868
Parathion	0.0207	0.5041	1.3093
Silibinin	0.0116	0.1916	1.6463
Cholesterol sulfate	0.0002	0.3329	1.5494
APC	0.0012	0.4954	1.4376
Citicoline	0.0254	1.7427	1.1961
3-Glucosyl-2,3',4,4',6-pentahydroxybenzophenone	0.0056	5.2503	1.4139
Macelignan	0.0014	0.4527	1.5044
Glycocholic acid	0.0170	6.9025	1.4201
d-Tocotrienol	0.0025	47.0597	1.6194
(3beta,5alpha,6beta,9alpha,22E,24R)-23-Methylergosta-7,22-diene-3,5,6,9-tetrol	0.0052	0.1621	1.6146
Ethyl tetradecanoate	0.0377	0.7688	1.1607
LysoPC(14:1(9Z))	0.0180	0.1315	1.5839
Epinephrine sulfate	0.0421	0.8642	1.1042
DHAP(18:0e)	0.0000	5.5699	1.4928
Yuccaol C	0.0037	7.4929	1.4398
Apo-12'-violaxanthal	0.0001	0.8950	1.6009
Phosphoric acid	0.0030	3.9946	1.6633
5-Ethoxysorgoleone 358	0.0104	0.3718	1.2974
Propylene glycol alginate	0.0001	3.4928	1.4270
Sebacic acid	0.0348	0.5900	1.2213
Zymonic acid	0.0433	0.6930	1.1229
(3beta,5alpha,9alpha,22E,24R)-3,5,9-Trihydroxy-23-methylergosta-7,22-dien-6-one	0.0191	0.3675	1.2484
Oxazepam	0.0005	0.7379	1.5385
Vignatic acid A	0.0051	0.2595	1.4317
Resveratrol	0.0003	0.6375	1.5877
Aromadendrin	0.0366	0.7630	1.1461

Methyl 5-hydroxyoxindole-3-acetate	0.0295	2.2318	1.1458
2-(a-Hydroxyethyl)thiamine diphosphate	0.0276	5.9558	1.3743
Protoporphyrin IX	0.0448	4.4060	1.2005
Musanolone C	0.0005	0.7709	1.5228
PC-M5'	0.0137	2.2671	1.2549
8-Hydroxy-deoxyguanosine	0.0042	0.5069	1.4485
PG(18:2(9Z,12Z)/22:6(4Z,7Z,10Z,13Z,16Z,19Z))	0.0496	0.4909	1.3012
PG(16:1(9Z)/18:0)	0.0125	7.3186	1.6286
BR-Xanthone B	0.0021	0.2798	1.3042
2-Chloro-5-methylmaleylacetate	0.0118	0.6457	1.2516
(3xi,7(11)Z)-7(11)-Copaene-4,12-diol	0.0167	0.1405	1.5667
Nevskin	0.0122	0.2005	1.4419
Pregnenolone	0.0002	5.9182	1.3110
1',2'-Dihydro-1,1'-dimethyl-2'-oxo-4,4'-bipyridinium(1+)	0.0000	0.4924	1.5871
Prostaglandin A2	0.0026	2.2341	1.4472

Table S3. 167 vital metabolites in Met group compared with DM group in hepatic metabolism.

**Metabolites**	**P-VALUE**	**FOLD CHANGE**	**VIP**
Adenosine	0.0033	2.9490	1.8460
Adenosine monophosphate (AMP)	0.0205	4.9769	1.8219
Hypoxanthine	0.0037	1.5418	1.8083
Norvaline	0.0436	1.4013	1.2484
5'-Inosinic acid	0.0393	6.0389	1.8481
Guanosine-5'-monophosphate	0.0328	5.4981	1.8679
Adenosine 5'-diphosphate	0.0023	2.0637	1.8795
Nicotinamide	0.0309	1.2274	1.4809
Glycerophosphocholine	0.0039	3.2948	1.8725
L-Pipecolic acid	0.0412	2.6319	1.2067
D-Pantothenic acid	0.0000	0.3897	2.2205
Dephospho coenzyme a	0.0302	3.2788	1.7747
Inosine	0.0001	3.6754	2.1247
Guanosine monophosphate	0.0262	6.0567	1.8357
Adenosine 3',5'-diphosphate	0.0058	1.6977	1.7559
ADP	0.0024	1.8788	1.8637
L-Malic acid	0.0345	1.1613	1.4445
Liquiritigenin 4'-[3-acetylapiosyl-(1->2)-glucoside]	0.0304	3.3399	1.6328
Xanthine	0.0080	0.5739	2.0538
L-Ascorbic acid	0.0023	1.5685	1.8717
3-Phosphoglyceric acid	0.0086	0.3101	1.7510
Uridine 5'-monophosphate	0.0278	10.1086	1.9565
Fumaric acid	0.0305	1.3118	1.5550
Glucosamine 6-phosphate	0.0051	2.1352	1.8558
Uric acid	0.0407	0.1508	1.9851

D-Glucose 6-Phosphate	0.0360	1.2269	1.4872
Guanine	0.0005	2.1343	1.9641
Maltotetraose	0.0278	3.7471	1.5966
S-Adenosyl-L-homocysteine	0.0009	2.4136	2.0222
4-Pyridoxic acid	0.0012	0.4709	1.8858
Lactose	0.0197	2.9507	1.5910
L-Threonine	0.0078	0.6655	1.6468
Hexadecanedioic acid	0.0138	0.6534	1.6928
Kojibiose	0.0263	2.7558	1.5276
Acetylphosphate	0.0272	0.3018	1.5742
Galactinol	0.0144	3.6901	1.5583
LPG(20:4)	0.0183	1.7154	1.8014
Oxypurinol	0.0224	0.7402	1.6716
LPE(20:4)	0.0447	1.3710	1.4460
Ononin	0.0028	3.7319	1.7750
5'-Methylthioadenosine	0.0017	3.7787	2.0115
D-Erythrose 4-phosphate	0.0006	10.1077	1.6721
PC(20:4/22:6)	0.0096	0.6095	1.6174
LPE(17:1)	0.0030	10.6696	2.1152
LPE(17:0)	0.0156	32.4627	2.1442
Salviaflaside methyl ester	0.0001	4.2031	2.0825
LPG(18:1)	0.0481	1.6334	1.5945
Riboflavin	0.0332	0.7236	1.5980
Dephospho-CoA	0.0209	3.1206	1.8584
8-Hydroxy-2'-deoxyguanosine	0.0005	2.7992	2.0024
3'-AMP	0.0430	3.4239	1.6815
D-Ribulose 5-phosphate	0.0026	1.8946	1.8449
Adenosine monophosphate	0.0031	2.5210	1.8259
Docosahexaenoyl Ethanolamide	0.0028	0.4809	2.0282
Thiamine monophosphate	0.0085	3.1674	1.9591
D-Xylose	0.0462	1.5573	1.3876
LPC(22:4)	0.0402	1.9695	1.4498
S-adenosyl-L-methionine	0.0224	4.8855	1.9507
Adenine	0.0080	1.9821	1.9687
Stearoylethanolamide	0.0451	1.3346	1.5502
Dehydroascorbic acid	0.0009	1.4977	1.9625
LPC(22:3)	0.0077	1.2842	1.7710
LysoPE(16:0/0:0)	0.0045	1.6843	1.8593
Epsilon-(gamma-Glutamyl)-lysine	0.0426	0.5411	1.6221
D-4'-Phosphopantothenate	0.0006	0.2449	2.0540
Graveoline	0.0193	2.2448	1.5893
Oleoyl glycine	0.0119	2.1278	1.1006
PC(2:0/17:0)	0.0212	3.9932	1.7670
3-Methylglutarylcarnitine	0.0176	6.6145	1.7861

Raffinose	0.0201	5.6406	1.7910
5-Methylcytidine	0.0483	6.3699	1.8687
LPE(22:6)	0.0163	1.3638	1.5656
Retinal	0.0345	2.2795	1.6415
PEtOH(16:1/20:4)	0.0001	8.3247	1.6668
8-HETE	0.0378	2.7545	1.7659
Dihydrotestosterone	0.0267	2.5250	1.8034
L-L-Homoglutathione	0.0028	1.9653	1.9142
Maleamic acid	0.0489	1.2559	1.3747
16(17)-EpDPE	0.0354	0.5271	1.4649
Pseudouridine	0.0171	1.3824	1.6964
N,N-Dimethylsphingosine	0.0077	1.2712	1.8641
L-Erythrulose	0.0435	1.5214	1.3846
ACRL Toxin II	0.0484	0.5438	1.4199
ACar(21:2)	0.0391	0.2474	1.3446
UDP-N-acetyl-alpha-D-galactosamine	0.0353	5.7494	1.9727
7-Methylguanine	0.0081	1.6118	1.4948
Stearoylglycine	0.0022	0.4114	1.9502
Glucosylisomaltol	0.0281	3.0642	1.4724
PEtOH(16:1/18:2)	0.0154	7.5177	2.0537
Ethylbenzene	0.0314	1.1550	1.5441
β-Alanine	0.0417	0.6960	1.4449
Amylopectin	0.0085	4.0253	1.5674
Trehalose	0.0325	2.1887	1.4625
2-Aminoheptanedioic acid	0.0098	6.2651	1.8860
Maltopentaose	0.0209	3.0664	1.4854
Uracil	0.0004	0.5192	2.0443
6-Phosphonoglucono-D-lactone	0.0113	1.9044	1.6839
Ethyl glucuronide	0.0067	6.9070	2.1152
Dihydro-3-(1-octenyl)-2,5-furandione	0.0208	2.3758	1.5103
1-Kestose	0.0233	6.8825	1.5928
FAHFA(16:0/3:0)	0.0425	0.6891	1.5117
Deoxyguanosine	0.0049	3.7796	1.7969
Lactodifucotetraose	0.0166	2.0213	1.8257
Dexamethasone	0.0074	6.6240	1.7988
1-Oleoylglycerophosphoinositol	0.0007	0.6979	1.9924
PC(2:0/20:3)	0.0340	2.6050	1.3754
Geniposide	0.0154	2.1139	1.9208
PI(18:1/20:4)	0.0293	0.6739	1.3937
5-Hydroxyflavone	0.0000	7.7477	1.7984
beta-D-Galactopyranosyl-(1->2)-[beta-D-galactopyranosyl-(1->4)]-D-galactose	0.0226	5.9881	1.8006
Acetylhomoserine	0.0320	1.4539	1.4893
LPA(16:1)	0.0082	2.0144	1.7722
PC(22:4(7Z,10Z,13Z,16Z)/18:2(9Z,12Z))	0.0304	0.7315	1.6082

4-(Trimethylammonio)butanoate	0.0162	2.3727	1.7952
5,10-Methylene-THF	0.0105	6.9716	2.0920
NAD	0.0162	1.2882	1.5751
Tetradecanedioic acid	0.0252	0.5393	1.6428
PI(20:4/20:4)	0.0017	0.3950	1.8933
3-beta-Glucosylcellotriose	0.0203	2.9883	1.4498
LPI(18:0)	0.0003	0.7560	2.0902
PG(16:3/22:6)	0.0428	2.0757	1.5019
Tryptophyl-Asparagine	0.0366	2.4871	1.5183
Piceatannol 4'-galloylglucoside	0.0062	1.8799	1.7958
PE(14:0/22:4(7Z,10Z,13Z,16Z))	0.0224	1.2405	1.5360
Adenosine phosphosulfate	0.0022	1.7666	1.9297
LPC(24:4)	0.0365	1.3580	1.2029
beta-Citraurin epoxide	0.0180	38.9425	2.1501
Monomethyl glutaric acid	0.0312	1.9765	1.5766
Ercalcitriol	0.0361	0.4264	1.8237
1-heptadecanoyl-glycero-3-phosphate	0.0018	2.9234	1.8714
Leukotriene B4	0.0094	0.6102	1.8653
Acetylleucine	0.0299	1.6626	1.4957
LysoPE(24:6(6Z,9Z,12Z,15Z,18Z,21Z)/0:0)	0.0387	2.2139	1.5186
Oxoadipic acid	0.0379	1.5428	1.3984
Uridine 5'-diphosphoglucuronic acid (UDP-D-glucuronate)	0.0021	3.2932	1.9554
rac-Normetanephrine	0.0152	0.7887	1.6673
Maltotriose	0.0213	6.2386	1.7587
Fenugreekine	0.0186	1.3346	1.6298
5,6-DHET	0.0119	0.5672	1.6883
Butyramide	0.0426	1.3925	1.4875
Gentiatibetine	0.0112	1.2520	1.6777
(R)-lipoic acid	0.0184	1.5879	1.6512
Rosmarinic acid	0.0032	3.4785	1.9627
Eicosapentaenoic acid	0.0060	0.6039	1.8436
Vitamin A	0.0400	2.6276	1.6993
CPA(18:1(9Z)/0:0)	0.0388	1.5332	1.6144
Inositol cyclic phosphate	0.0012	1.7205	1.8987
14R,15S-EpETrE	0.0057	0.5149	1.8034
Methyl 2,3,6-tri-O-galloyl-beta-D-glucopyranoside	0.0005	2.7497	1.9743
Tyrosyl-Histidine	0.0378	2.1557	1.2299
6-(alpha-D-Glucosaminyl)-1D-myo-inositol	0.0230	2.4409	1.5921
3'-Sialyllactose	0.0491	3.9066	1.4672
LysoPA(16:0/0:0)	0.0133	1.2627	1.6459
Eujambolin	0.0115	0.1532	1.8121
N-cis-Feruloyltyramine	0.0018	2.2388	1.9758
APC	0.0428	0.6779	1.4322
Macelignan	0.0129	0.5547	1.5944

Leukotriene C4	0.0118	0.6644	1.8476
Yuccaol C	0.0270	5.6156	1.6784
2,5-Dihydro-2,4,5-trimethyloxazole	0.0016	0.8256	1.8797
Palmitoyl Serinol	0.0057	1.2150	1.7526
Resveratrol	0.0248	0.7253	1.5672
CDP-Ethanolamine	0.0238	2.1163	1.7569
Physagulin F	0.0467	0.7379	1.4979
Nevskin	0.0266	0.4317	1.6586
1',2'-Dihydro-1,1'-dimethyl-2'-oxo-4,4'-bipyridinium(1+)	0.0000	0.4214	2.2265
LysoPE(22:5(4Z,7Z,10Z,13Z,16Z)/0:0)	0.0197	1.7434	1.6986

Table S4. 139 vital metabolites in AX400 group compared with DM group in hepatic metabolism.

**Metabolites**	**P-VALUE**	**FOLD CHANGE**	**VIP**
Hypoxanthine	0.0016	1.7063	2.0406
L-Phenylalanine	0.0324	0.7969	1.5328
D-Proline	0.0149	0.7430	1.8102
D-Pantothenic acid	0.0004	0.4445	1.9049
Dephospho coenzyme a	0.0385	2.1502	1.7857
Inosine	0.0019	3.1488	2.1082
Adenosine 3',5'-diphosphate	0.0363	1.6664	1.7135
Xanthine	0.0030	0.5631	2.0246
Guanosine diphosphate	0.0389	1.8966	1.2318
Uric acid	0.0377	0.1301	2.0853
DL-Phenylalanine	0.0052	0.7215	1.8093
Guanine	0.0022	2.6160	1.9863
4-Pyridoxic acid	0.0008	0.4507	2.0367
5-Aminovaleric acid	0.0030	1.0963	2.0205
FAHFA(22:4/22:3)	0.0419	0.5194	1.2146
FAD	0.0312	1.2219	1.5463
Ergothioneine	0.0077	1.4366	1.8517
Valyl-Leucine	0.0462	0.6521	1.4674
Oxypurinol	0.0079	0.6452	1.9144
Ononin	0.0122	3.2759	1.6687
FAHFA(20:2/22:3)	0.0356	0.6246	1.4926
5'-Methylthioadenosine	0.0003	2.9195	2.0370
D-Erythrose 4-phosphate	0.0001	9.6211	1.7796
LPE(17:1)	0.0137	12.3206	2.1495
LPE(17:0)	0.0024	33.0937	2.2639
Salviaflaside methyl ester	0.0007	4.1152	2.1042
Dephospho-CoA	0.0240	2.2843	1.8907
8-Hydroxy-2'-deoxyguanosine	0.0046	3.2687	1.9859
3'-AMP	0.0134	2.0883	1.9162
PC(22:6e/7:0)	0.0417	2.0505	1.6030

Docosahexaenoyl Ethanolamide	0.0241	0.6190	1.7301
Thiamine monophosphate	0.0027	2.1702	1.9682
S-adenosyl-L-methionine	0.0005	3.0893	1.9753
Arachidonic acid	0.0468	0.7781	1.4292
LPC(15:0)	0.0366	1.3613	1.6377
LPC(22:3)	0.0479	1.1335	1.5492
N6-Carbamoyl-L-threonyladenosine	0.0235	0.8104	1.6657
Epsilon-(gamma-Glutamyl)-lysine	0.0411	0.4812	1.9530
D-4'-Phosphopantothenate	0.0034	0.4116	1.9101
4-Chloro-3,5-dimethoxybenzyl alcohol	0.0096	1.0748	1.8847
Oleoyl glycine	0.0356	2.2404	1.2070
Symmetric dimethylarginine	0.0185	0.6631	1.6086
Retinal	0.0341	1.6616	1.8512
PEtOH(16:1/20:4)	0.0062	6.5372	1.5094
8-HETE	0.0013	1.6451	2.0069
Dihydrotestosterone	0.0269	1.4294	1.6360
L-L-Homoglutathione	0.0269	1.3451	1.5391
Pseudouridine	0.0088	1.4764	1.8973
ACar(20:2)	0.0041	0.3015	1.8586
2-Furanmethanol	0.0116	1.8313	1.8577
9,10-epoxyoctadecanoic acid	0.0173	0.5480	1.5778
Rishitin	0.0487	1.1569	1.6400
LPC(20:4)	0.0174	0.3108	1.9794
ACRL Toxin II	0.0128	0.5393	1.8089
UDP-N-acetyl-alpha-D-galactosamine	0.0156	2.3877	1.9040
PEtOH(16:1/18:2)	0.0178	9.7610	2.1270
L-Valine	0.0114	0.6842	1.7128
LPC(18:3)	0.0306	1.3795	1.5598
p-Mentha-1,8-dien-7-ol	0.0130	1.0658	1.7990
FAHFA(18:1/3:0)	0.0481	0.3736	1.5726
Sciadonic acid	0.0060	30.6505	2.2387
2-Aminoheptanedioic acid	0.0013	5.3079	1.9584
N-Arachidonoyl GABA	0.0003	9.7043	2.0417
Uracil	0.0005	0.5862	2.1515
2,4,6-Octatriynoic acid	0.0329	0.8181	1.5068
Polyoxyethylene (600) monoricinoleate	0.0279	1.4362	1.5372
Coenzyme A	0.0291	2.5732	1.6996
Ethyl glucuronide	0.0001	3.8125	2.1665
(Â±)8(9)-EET Ethanolamide	0.0033	7.9762	2.1278
Astragalin	0.0301	0.3854	1.5693
Austroinulin	0.0104	51.8625	2.1471
FAHFA(16:0/3:0)	0.0209	0.6340	1.7112
9,10-DHOME	0.0479	0.6394	1.4334
Lactodifucotetraose	0.0162	1.6446	1.7943

Dexamethasone	0.0211	5.7225	1.8611
3-Methyl-5-pentyl-2-furanundecanoic acid	0.0057	10.1230	1.7475
PA(2:0/18:0)	0.0475	0.1887	1.9575
Cappariloside A	0.0137	0.6703	1.6489
5-Hydroxyflavone	0.0010	7.9554	1.8272
3-Hydroxy-carbofuran	0.0397	0.5193	1.6801
PE(18:1/20:4)	0.0347	0.7805	1.5646
13S-hydroxyoctadecadienoic acid	0.0211	0.6603	1.6961
Dioscorine	0.0257	1.2135	1.6464
5,10-Methylene-THF	0.0103	4.0234	2.2153
Adrenosterone	0.0338	0.2510	2.0675
Perilloside C	0.0421	0.4924	1.1823
Mesaconic acid	0.0457	0.4890	1.8026
Cannabidiol	0.0087	0.5157	1.8513
D-Pinitol	0.0113	0.5224	1.7660
PI(20:4/20:4)	0.0078	0.5809	1.8754
Isosalsolidine	0.0365	0.4326	1.1515
4-Oxo-2-nonenal	0.0310	2.1303	1.7130
LPI(20:3)	0.0123	0.5981	1.8951
Piceatannol 4'-galloylglucoside	0.0070	2.0322	1.8992
Pantetheine 4'-phosphate	0.0023	2.3051	2.2023
beta-Citraurin epoxide	0.0038	39.4845	2.2851
L-phenylalanyl-L-hydroxyproline	0.0482	2.8403	1.9377
1-heptadecanoyl-glycero-3-phosphate	0.0301	1.9227	1.5057
LysoPI(18:0/0:0)	0.0307	0.6852	1.5247
Arginyl-Methionine	0.0347	2.1860	1.6751
Leukotriene B4	0.0474	0.7041	1.5596
rac-Normetanephrine	0.0075	0.7961	1.8793
dIMP	0.0287	0.5492	1.6192
2-hydroxydesipramine	0.0273	0.7256	1.5852
Undecanoic acid	0.0450	0.6577	1.4828
3,4-Dihydrocoumarin	0.0191	0.7109	1.6608
5,6-DHET	0.0022	0.5293	1.9152
Butyramide	0.0427	1.4838	1.7493
Cis-zeatin-O-glucoside	0.0088	2.6425	1.9593
Gentiatibetine	0.0098	1.2424	1.8140
xi-3-Hydroxy-5-phenylpentanoic acid O-beta-D-Glucopyranoside	0.0022	1.5494	1.8977
10-Hydroxymelleolide	0.0497	0.5658	1.5732
Calabaxanthone	0.0369	2.2481	1.3345
Rosmarinic acid	0.0083	3.4301	1.9806
Behenic acid	0.0151	1.8616	1.1793
Octadecanedioic acid	0.0025	2.2214	1.9503
Eicosapentaenoic acid	0.0215	0.6990	1.5797
Vitamin A	0.0103	1.6269	1.8265

14R,15S-EpETrE	0.0004	0.6290	2.1127
(3beta,22R,23R,24S)-3,22,23-Trihydroxystigmastan-6-one	0.0223	0.6633	1.7517
Methyl 2,3,6-tri-O-galloyl-beta-D-glucopyranoside	0.0004	2.8560	2.0236
N-cis-Feruloyltyramine	0.0093	2.2083	1.9375
Demethoxyfumitremorgin C	0.0196	2.2781	1.5186
cis-3-Hexenyl lactate	0.0148	0.6982	1.8249
Diacetone alcohol	0.0496	0.8587	1.4498
Citicoline	0.0033	2.6624	2.0304
Macelignan	0.0307	0.5506	1.5838
d-Tocotrienol	0.0080	21.2782	2.1100
Leukotriene C4	0.0117	0.5856	1.7457
Epinephrine sulfate	0.0266	0.8415	1.5571
(3S,5R,6R,7E)-3,5,6-Trihydroxy-7-megastigmen-9-one	0.0089	1.3436	1.8318
Zymonic acid	0.0222	1.3761	1.5921
Resveratrol	0.0002	0.5222	2.0647
Aromadendrin	0.0321	0.7712	1.5187
CDP-Ethanolamine	0.0024	1.9955	1.9728
Protoporphyrin IX	0.0256	2.2178	1.6208
Cyclotetradecane	0.0046	1.1004	1.8867
1',2'-Dihydro-1,1'-dimethyl-2'-oxo-4,4'-bipyridinium(1+)	0.0003	0.4755	1.9496
Ganolucidic acid E	0.0010	0.5354	1.9312
